# The complete mitochondrial genome of *Poropuntius huangchuchieni* (Cyprinidae)

**DOI:** 10.1080/23802359.2020.1719914

**Published:** 2020-02-11

**Authors:** Chenru Yang, Yan Zhang, Hongwei Wang

**Affiliations:** a National Demonstration Center for Experimental Fisheries Science Education, Shanghai Ocean University, Shanghai, China;; b Key Laboratory of Aquatic Genomics, Ministry of Agriculture, and Beijing Key Laboratory of Fishery Biotechnology, Chinese Academy of Fishery Sciences, Beijing, China

**Keywords:** *Poropuntius huangchuchieni*, Yunnan Plateau, mitochondrial genome

## Abstract

We sequenced the mitogenome of *Poropuntius huangchuchieni* from Yunnan Plateau. The mitogenome was 16,554 bp in length and comprised 13 protein-coding genes, 22 transfer RNAs, and 2 ribosomal RNAs. The base composition included 32.3% for A, 25.8% for T, 15.6% for G, and 26.3% for C, respectively. The gene order was identical to other published Cyprinidae species. The phylogenetic analysis showed that *P. huangchuchieni* was close to *Puntius tetrazona*, a *Puntigrus* fish in Cyprinidae family.

*Poropuntius huangchuchieni* is a freshwater fish and belongs to the *Poropuntius* genus, Cyprinidae family (Ding et al. 2013). It is widely distributed in Mekong and Red Rivers of Yunnan Plateau in China. Examining the phylogeography of this species from different drainages would be applied to evaluating the effects of the geologic movement and climate change on the drainages in Yunnan Plateau (Wu et al. 2013). The mitogenome of *P. huangchuchieni* would help study the phylogeographic pattern of this fish and determine the taxonomic relationship between this species and other Cyprinidae fish.

Here we report its mitochondrial genome (GenBank accession: MN723896.1). One specimen of *P. huangchuchieni* was collected from the Lancang River, Xishuangbanna, Yunnan Province, China (21°48′ N and 101°57′E) and was stored in the fish specimen room of Center for Applied Aquatic Genomics, Chinese Academy of Fishery Sciences (specimen Accession number: CAAG-2019-1219). Total genomic DNA was extracted with the DNeasy Tissue Kit (Qiagen, Valencia, CA) and then the mitochondrial genome was sequenced and assembled following Xue et al.’s method (Xue et al. 2013), and then annotated using MitoAnnotator (Bernt et al. 2013).

The complete mitogenome of *P. huangchuchieni* is 16,554 bp in length, including 13 protein-coding genes (PCGs), 22 transfer RNAs, and 2 ribosomal RNAs. Among the 13 PCGs, only *COX1* uses GTG as the start codon and the rest genes initiate with the start codon ATG. The most common stop codons are TAA (*COX1*, *ATP6*, *COX3*, *NAD4L*, *NAD5*, and *NAD6*) and TAG (*NAD1*, *NAD3*, and *ATP8*). The rest four PCGs (*COX2*, *NAD2*, *NAD4*, and *COB*) end with the incomplete stop codon T (Boore 1999). The overall nucleotide composition was estimated to be 32.3% of A, 25.8% of T, 15.6% of G, and 26.3% of C, with a slightly higher A + T content (58.1%).

Based on the PCGs encoded in *P. huangchuchieni* and the other nine Cyprinidae fish, a phylogenetic analysis (Figure 1) was carried out using the MEGA7 package (Kumar et al. 2016) with the parameters of maximum-likelihood (ML) analysis, Jones–Taylor–Thornton model, and 1000 bootstrap replicates. The analysis revealed that *P. huangchuchieni* was clustered with *Puntius tetrazona*, a fish in *Puntigrus* genus, Cyprinidae family. The mitogenome resource will contribute to study the biogeography and diversification of *P. huangchuchieni* in Yunnan Plateau.

**Figure 1.**
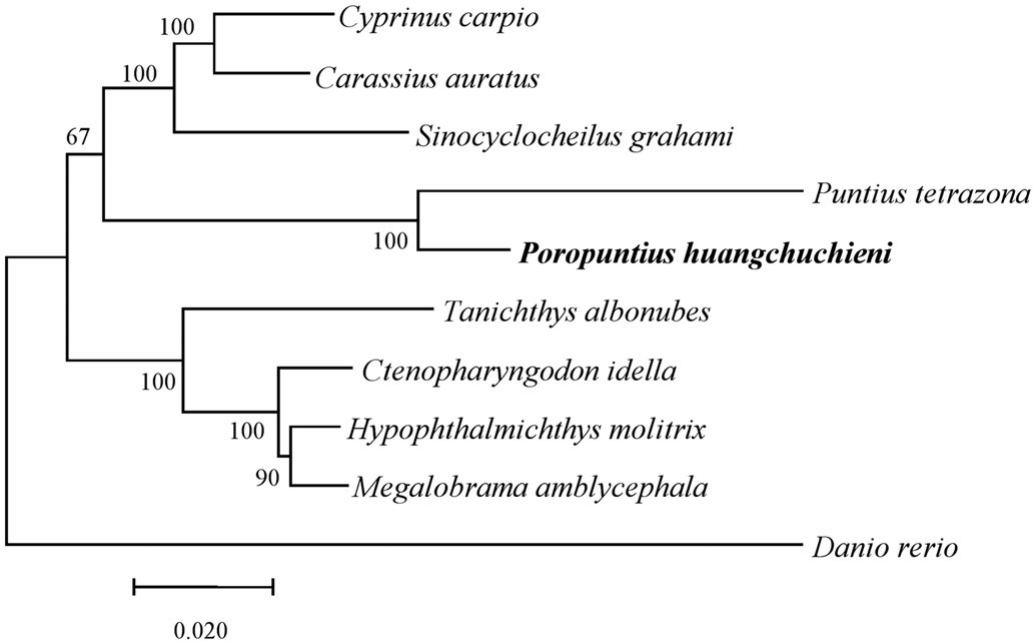
A phylogenetic tree of *P. huangchuchieni* and the other nine species. The species used are as follows: *Cyprinus carpio* (NC_001606.1), *Carassius auratus* (NC_002079.1), *Sinocyclocheilus grahami* (NC_013189.1), *Puntius tetrazona* (NC_010110.1), *Tanichthys albonubes* (NC_015539.1), *Ctenopharyngodon idella* (NC_010288.1), *Hypophthalmichthys molitrix* (NC_010156.1), *Megalobrama amblycephala* (NC_010341.1), and *Danio rerio* (NC_002333.2). The number at each branch is the bootstrap value of ML analysis.
